# The Digital Twin Paradigm Applied to Soil Quality Assessment: A Systematic Literature Review

**DOI:** 10.3390/s23021007

**Published:** 2023-01-15

**Authors:** Letícia Silva, Francisco Rodríguez-Sedano, Paula Baptista, João Paulo Coelho

**Affiliations:** 1 Research Center for Digitization and Intelligent Robotics (CeDRI), 5300-253 Bragança, Portugal; 2 Robotics Group, Engineering School, University of León, Campus de Vegazana, 24071 León, Spain; 3 Laboratório para a Sustentabilidade e Tecnologia em Regiões de Montanha (SusTEC), 5300-253 Bragança, Portugal; 4 Instituto Politécnico de Bragança, Campus de Santa Apolónia, 5300-253 Bragança, Portugal; 5 Mountain Research Center (CIMO), Instituto Politécnico de Bragança, Campus de Santa Apolónia, 5300-253 Bragança, Portugal

**Keywords:** precision agriculture, digital twins, soil quality, systematic review

## Abstract

This article presents the results regarding a systematic literature review procedure on digital twins applied to precision agriculture. In particular, research and development activities aimed at the use of digital twins, in the context of predictive control, with the purpose of improving soil quality. This study was carried out through an exhaustive search of scientific literature on five different databases. A total of 158 articles were extracted as a result of this search. After a first screening process, only 11 articles were considered to be aligned with the current topic. Subsequently, these articles were categorised to extract all relevant information, using the preferred reporting items for systematic reviews and meta-analyses methods. Based on the obtained results, there are two main conclusions to draw: First, when compared with industrial processes, there is only a very slight rising trend regarding the use of digital twins in agriculture. Second, within the time frame in which this work was carried out, it was not possible to find any published paper on the use of digital twins for soil quality improvement within a model predictive control context.

## 1. Introduction

According to [1], the underlying concept of digital twins was born after a presentation of M. Grieves at the University of Michigan in the early 2000s. Initially referred to as the “Mirrored Spaces Model”, this paradigm was presented in the context of product lifecycle management, where each system could be decomposed in two components: the physical system and its mirrored virtual entity where all the information about the system’s states could be reached [2,3]. Later, in [4], M. Grieves alluded to the same concept using the alternative term “digital twin”, which was put forward by J. Vickers, a NASA technologist in advanced materials [5]. For this reason, many authors credit NASA, the United States of America National Aeronautics and Space Administration, with coining the term “digital twin” [3,6,7]. Grieves and Vickers worked together on adapting the concept of digital twins in the manufacturing sector to improve product lifecycle management [8]. Recently, in the Industry 4.0 technological umbrella, digital twins was presented as a way to increase productivity, efficiency, and adaptability [3,9,10,11,12,13]. The use of digital twins has been reported in the manufacturing process [14] of the automotive and energy [15] or even aerospace and health [13] industries.

At its basic level, digital twins can be seen as realistic virtual representations (digital replicas) of entities with physical or logical existence, such as machines or processes. This virtual representation is accomplished by modelling the processes and assets using a broad range of different mathematical models and feeding them with massive amounts of real-world data. At the present, those data sets can be obtained through real-time data acquisition systems based on Internet of Things (IoT) technologies [16,17]. A digital twin model presents a data flow that automatically fluxes from the virtual entity to the physical entity, and the same process happens in the opposite direction, from the physical entity to the virtual entity [13,18].

The implementation of the digital twin enables experimental process testing that facilitates the discovery of optimisation points and process simulation. Moreover, the digital system’s profile can be seen as a fundamental component in a closed-loop control strategy, where the information provided by the digital twin may lead to physical actions that drive changes in the manufacturing processes. In addition, it also enables a complete understanding of the system’s operating mechanisms and contributes to increasing agility and robustness in response to disturbances [19,20]. Digital twins can also be used to investigate the status history and simulate future habits. In this way, they are implemented as a way of answering hypothetical questions and providing spontaneous revelations [21].

Extrapolation from the industrial realm to primary sectors, such as agriculture, is a natural step to take. Indeed, for agricultural processes, the same goals of productivity increase and product quality control are typical concerns. For this reason, agriculture virtualisation comes as a natural response since it seems to be a direct proportional relationship between productivity and agriculture digitalisation [22,23,24]. The societal relevance of this approach is translated by the amount of funding provided for research in this area. Among many others, the IoF2020 European project is an example where digital twins are employed in the agri-food area [13,25].

As in many other sectors, agriculture has seen an increase in data collection for decision support. For example, through the deployment of local sensors and drones to access weather data and satellite images from remote servers, growers now have access to more information about the climate, soil, and crop vegetative states. In some way, these conditions converge to promote and speed up the integration of digital twins in agricultural processes. However, despite all this potential, their use in this context is still at an early stage of deployment [13,26,27]. There are several reasons that can be pointed out regarding why the use of digital twins in agriculture has not yet taken a quantitative leap. On one hand, agricultural processes are usually more complex than industrial processes. This complexity is not only due to the high dimensionality of the data but also to the fact that many of the variables involved, which strongly condition the behaviour of the processes, are stochastic in nature and cannot be manipulated or controlled. Moreover, the large area in which agriculture processes take place, in conjunction with the heterogeneous conditions found along those terrains, requires spacial and temporal data resolutions that are not technically and economically feasible. In this frame of reference, the present paper aims to present the results concerning a systematic literature review on digital twins in the context of precision agriculture.

In general, the concept of precision agriculture is tightly connected to crop management paradigms, where a layer of information technology is added on top of standard production processes. Spatial and temporal information, obtained from a myriad of different sources, can be integrated into machine learning and artificial intelligence algorithms to attain surgical control over all operational aspects of the production activities. With this new approach, it is expected to reduce the input of chemicals (for weed control, plant protection against pests and diseases, and fertilisation) and the consequent negative effects on soil health and overall biodiversity while maintaining crop profitability and good-quality food. Indeed, the information provided by these new approaches would support farmer’s decision making regarding site-specific management practices, thus allowing them to use resources more efficiently and with less environmental impact.

The focus will be narrowed down to the published research that targets the use of such an approach for promoting the soil quality. Soil quality and soil health are often used synonymously, and are two terms very difficult to define due to the extreme complexity of the soil ecosystem [28]. A healthy soil is generally defined as a soil with capacity to provide ecosystem services for all forms of life, while soil quality concerns the capacity of a specific soil to sustain a particular use, such as crop production [29]. Therefore, soil health/quality are two important parameters that affect the production of resources in agricultural fields. Recently, both soil health/quality have been receiving increasing interest from the scientific and political community. Indeed, in recent decades, soils around the world have been subject to severe and quick degradation processes, mostly due to climate change, land-use changes, and the implementation of unsustainable agricultural practices, such as the use of synthetic chemical fertilisers and soil tillage, among others [30]. These practices have triggered a series of cascading effects within the ecosystem, such as soil erosion, nutrient and moisture depletion, degradation, and loss of biodiversity [30]. It is therefore of primary importance to act in order to improve and/or restore the “health” and “quality” of soils. In this regard, precision agriculture and digital agriculture can play an important role. Soil quality is a very broad and generic term as it can be considered from a chemical, physical and biological perspective, or any combination of the three. In the current context of this work, soil quality will be measured based on the distance between the ideal chemical and physical characteristics for a healthy soil in olive oil production, in addition to the effectively observed characteristics. Regarding physical quantities, we speak, for example, of the temperature and relative humidity of the soil at different depths and electro-conductivities. From a chemical point of view, the pH and dissolved oxygen will be considered. Moreover, the concentration of nitrogen, phosphorus, and potassium will also be measured. Effects due to mechanical activities, such as ploughing and tilling, which cannot be performed automatically, will not be taken into consideration.

In this work, particular interest will be directed to situations where the dynamic behaviour of the process, translated by a digital twin, is included in a closed-loop control strategy based on predictions generated by that model.

This systematic review has targeted publications made since the year 2016 using five different databases. Details regarding the methodology applied to carry out the systematic review of the literature, the planning, and the conduction process are provided in Section 2. Section 3 presents the results and their respective discussion. After an analysis of the selected articles and the application of the selection criteria, a rigorous discussion of the subject is presented. Finally, in Section 4, we highlight our final conclusions and remarks.

## 2. Applied Methodology

A systematic literature review (SLR) is a tool used to evaluate and interpret all accessible and relevant research for the occurrences of keywords of interest and/or search questions. Using the systematic literature review method instituted by Kitchenham, this work was elaborated [27,31,32,33]. The paths to developing an SLR are divided into three main steps: planning, conducting, and reporting the review. Each of the phases plays an important role in the final review. The SLR’s mission is to present an assessment of a research topic by applying a methodology seen as reliable, accurate, and able to be audited [34,35,36,37].

It is very important to carry out a preliminary search in a database available on the internet to identify if there is an SLR with an identical research topic. This should be performed before starting SLR planning. A new search would be unnecessary if a similar SLR already exists [38,39,40]. In the case of this SLR, several results were found for the theme “Digital Twins in Agriculture”. Modifications were made so that the topic became more restricted. For the new theme, “Digital Twins in Precision Agriculture for Soil Quality Improvement”, no identical results were found.

### 2.1. Review Planning

The SLR planning is divided into the definition of the search questions; the elaboration of the PICOC; the selection of keywords and synonyms; the determination of inclusion and exclusion criteria for articles from this systematic literature review; the creation of the search string; the choice of search sources; quality assessment; and the data extraction form.

#### 2.1.1. Definition of Search Questions

In review planning, the process of identifying and defining the review execution modes is determined, so that the review is completely replicable and traceable [36]. In the first step, it is necessary to specify the research question to be investigated. The research questions (RQs) are presented in Table 1.

#### 2.1.2. Preparation of the PICOC Method

In the second stage, after the determination of the QRs, the PICOC methodology was applied to define the purpose of the review. This methodology helps in the article analysis process and was developed and described by Petticrew and Roberts [38]). Its characterisation is shown as:

- Population (P): Who?
- Intervention (I): What and how?
- Comparison (C): What to compare?
- Result (O): What are the final goals you seek to achieve?
- Context (C): What are the contexts?

The PICOC method, previously defined, will be used in this search, and is presented in Table 2.

#### 2.1.3. Selection of Keywords and Synonyms

In the third step, the keywords were defined. Table 3 presents the results of the keywords and their corresponding synonyms, in addition to relating them to the PICOC method.

#### 2.1.4. Inclusion and Exclusion Criteria

To define the relevant articles and relate them to the research questions, the inclusion (IC) and exclusion (EC) rules were established. If the article meets any of the following criteria, it must be included or withdrawn from the study. The inclusion criteria are presented in Table 4.

The exclusion criteria are presented in Table 5.

The articles were found and extracted from the libraries, to which the inclusion (IC) and exclusion (EC) criteria were applied. In this step, it is necessary to read the abstract and the keywords to define the relevant articles. Finally, they were accepted to integrate the following steps of the final systematic review under study.

#### 2.1.5. Quality Criteria

In this stage, quality criteria are applied based on questions of verification and an analysis of the selected paper quality. The article needs to be read completely, and the questions must be very specific, with each one having a specific score. The quality questions (QQ) are presented in Table 6.

The answer can have three different values: 1.0 (highest score) if the question is completely answered, 0.5 (medium score) if the question is partially answered, and 0.0 (lowest score) if the question is not answered.

All questions can be answered with “yes” or “no”. The first three questions are more comprehensive (QQ1, QQ2, QQ3). The following questions are more specific (QQ4, QQ5, QQ6, QQ7, QQ8, QQ9).

The questions QQ4, QQ5, QQ6, QQ7, QQ8, and QQ9 have high relevance in determining the quality of the publications under study because they answer the main topics of interest in this systematic review. The sum of the maximum score of these questions corresponds to a score of 6.0.

Based on the quality criteria, the cut-off score is 6.0 for any article. In this way, all publications that have a grade greater than 6.0 are added to the final SLR. Furthermore, those with a lower score are excluded. Likewise, the maximum score for any article was defined as 9.0. Therefore, no article can score higher than that. The results of this quality criteria step are presented in Table 3 of Section 3.

#### 2.1.6. Data Extraction Form

The final step of SLR planning is data extraction, in which new data extraction questions must be created to determine meaningful information and extract it. Data extraction issues (DQ) are determined, as follows, in Table 7.

After the data extraction step, the results obtained were organised in a table format and presented in the results section (Section 3) in Table 4. All the classified articles must present a score equal to or greater than 6.0.

### 2.2. Conduction

**Figure 1:**
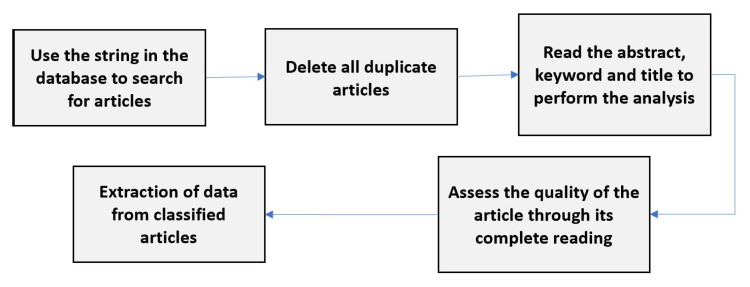
Conduction process.

Subsequently, in the planning stage, the process called driving must be carried out. This was carried out following the PRISMA method, which characterises and describes in detail the phases of the driving process. Figure 1 illustrates the process of performing SLR.

Regarding conduction stages, they are broken down into identification, triage, eligibility, and inclusion.

Upon identification, articles are discovered in each search source using the search string and are saved. Subsequently, repeated articles were removed. In triage, only the title, abstract, and keywords were read in each publication. Then, inclusion and exclusion criteria were applied, and publications that are not classified by the established criteria are also excluded. For the remaining publications, eligibility was applied to the defined quality issues. Each publication, read in its entirety, had a score assigned. Furthermore, those who do not obtain a minimum score above the pre-established limit (6.0) were excluded. Upon inclusion, publications that obtained high value were approved and classified for the final SLR. Eventually, data extractions were performed based on extraction questions.

The instrument used to complete this systematic literature review was the program called “PICO Portal” *©* (https://picoportal.org/about-us/ (accessed on 6 January 2022)).

In this way, by using this tool, it is possible to organise the steps more easily, plan the review, import the work, and ask questions. Finally, a final report on the review and its main features was prepared, with results being presented in the Results section (Section 3).

#### Creation of the String and Selection of Search Sources

In this stage, the search strings are created. It is secondly expressed as an equation that evidences all the main terms of the search. To find relevant publications on the topic, this string needs to be tested in each one of the databases. Depending on the website, the search string may vary and require specific uses of characters.The search string created for this systematic review is presented:

*(“digital twin*∗*”) AND (agri*∗ *OR crop*∗ *OR farm*∗*) AND (soil OR land OR field OR “field*∗ *management*∗*” OR “soil quality*∗*”)*:

These databases are well-known in this field of research: Springer Link, Association for Computing Machinery, ISI Web of Science, Institute of Electrical and Electronics Engineers, and Scopus. All selected search sources are important and known in the research area, especially in the areas of technology. Furthermore, they are very relevant to the research topic developed in this literature review. Table 8 shows the search phrase used for all digital libraries.

- Springer Link (SL): From the Springer Link library (https://link.springer.com/, accessed on 30 May 2022).
  In the first search, the query string was used in the simple search bar of the site, and many results were found. It is necessary to select the option “include content for viewing only”, and also the filters “content type: article” and “discipline: engineering”.
- Biblioteca Digital—Association for Computing Machinery (ACM): From the Digital ACM library (https://dl.acm.org/, accessed on 30 May 2022).
  The advanced search feature was used and the search string used was highlighted. Furthermore, the “Research Article” filter was applied.
- ISI Web of Science (WoS): From the ISI Web of Science library (https://access.clarivate.com/, accessed on 30 May 2022).
  The query was performed in the search tab and a search string was added.
- Digital Library—Institute of Electrical and Electronics Engineers (IEEE): From the IEEE Digital Library (https://ieeexplore.ieee.org, accessed on 30 May 2022).
  The search was performed in the simple search bar tab on the site, and the option “ALL” was selected. Furthermore, we added the search strings in the tab.
- Scopus: From the Scopus library (https://www.scopus.com/, accessed on 30 May 2022).
  An advanced search was performed. The following filters were applied: “article”, “engineering”, “keyword: digital twin”.

## 3. Results and Discussion

In this section, all the data extracted during the systematic literature review process, through planning and conduction, is presented. Then, the results obtained are shown, followed by respective analyses and discussions.

### 3.1. Results

**Figure 2:**
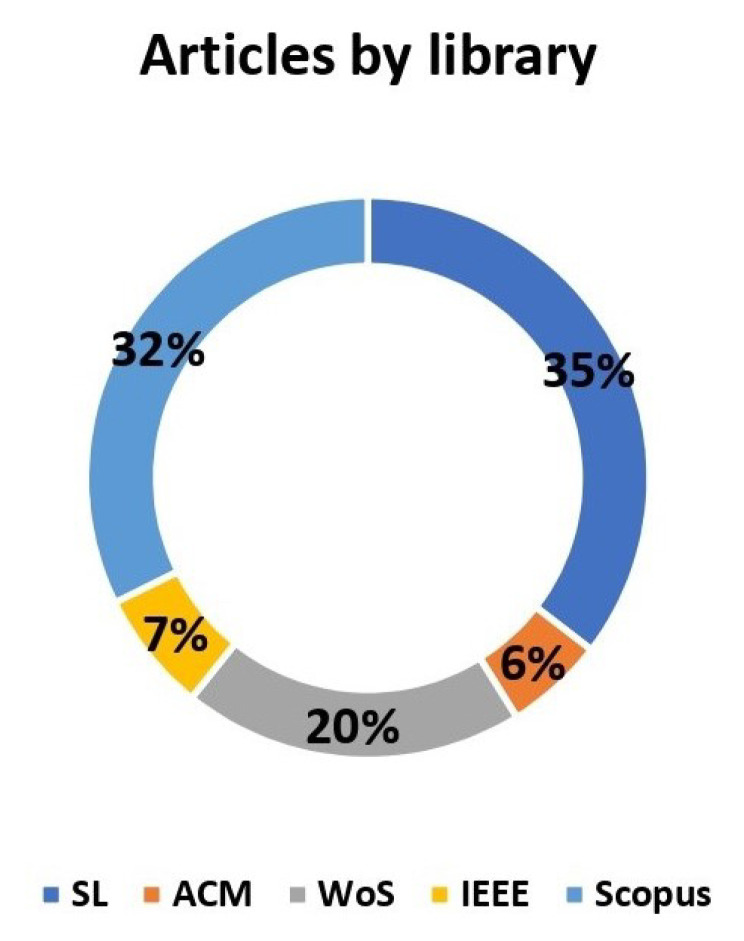
Percentage of articles by libraries (at the end of the Review).

In this section, the results obtained in the planning and conduction process are presented. Table 9 presents the number of articles extracted from each library at the beginning of the review process. Furthermore, it shows the percentage of articles selected (in the end) by a library. Figure 2 shows the percentage of articles selected by a library at the end of the review.

In the identification step, the search is performed in five databases using the search string from the obtained results. A total of 158 articles were found, and we removed 28 duplicates.

In the triage stage, we considered the 130 remaining articles, and after the inclusion and exclusion criteria were applied, 109 articles were excluded. In the eligibility stage, the 21 remaining articles were read in full before a quality assessment was performed (quality questions), in which 10 articles were excluded.

**Figure 3:**
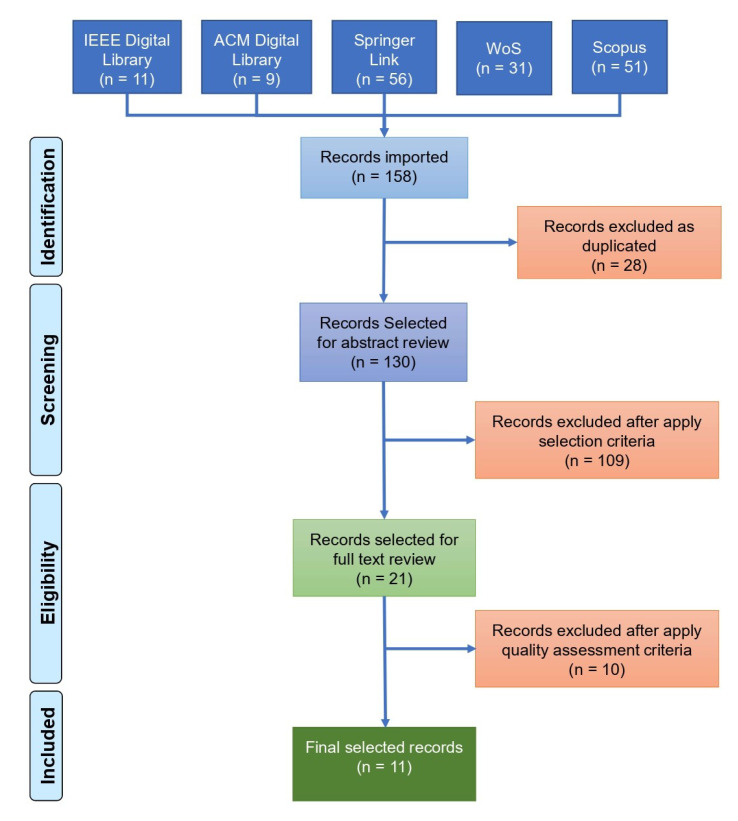
Results of the conduction process. Source: author.

In inclusion, as a result of the filters applied in the previous phases, a total of 11 articles were selected to integrate the systematic literature review and data extraction. Thus, these articles met all the criteria evaluated. The entire conduction process is shown in the diagram of Figure 3.

Table 10 presents the results of the 21 articles selected by the quality assessment, of which 11 were classified for the data extraction stage. Each of the articles that scored more than 6.0 in the answers to the quality questions moved on to the next phase, which contained the most important scoring questions (pre-set along with the quality criteria). Ten articles were excluded for presenting scores below the established limit.

Table 11 presents the data referring to two stages of the SLR. The first column corresponds to the years in which the articles were published. The second column corresponds to the number of articles selected and the result of the first phase of the systematic review. Furthermore, the third corresponds to the number of articles accepted and the result of the quality criteria stage.

**Figure 4:**
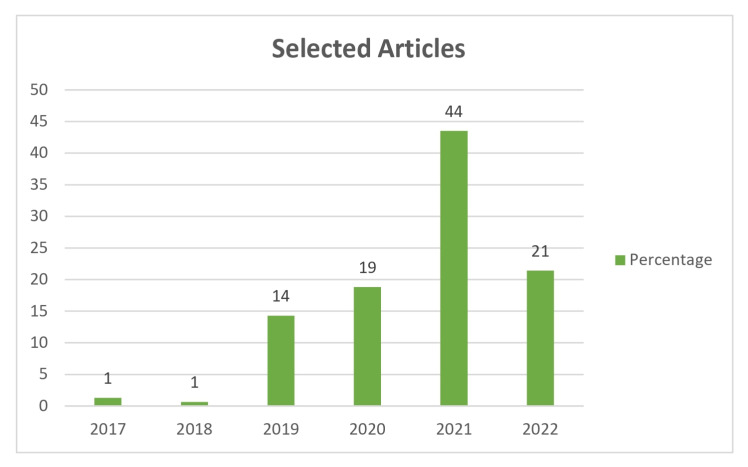
Percentage of articles selected.

**Figure 5:**
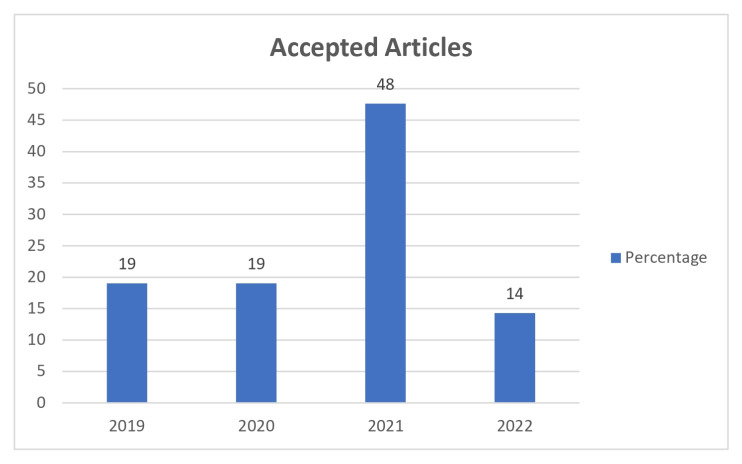
Percentage of articles accepted.

Figure 4 symbolises the percentage of articles selected per year through the use of search phrases. Figure 5 symbolises the percentage of articles accepted per year through the application of quality criteria. By analysing these figures, it can be seen that the articles found are very recent.

From the scores determined in the previous step, it is possible to calculate the statistical values of the median and average and determine the maximum and minimum values. Furthermore, this also indicates the cut-off score. Table 12 presents the statistical calculations.

**Figure 6:**
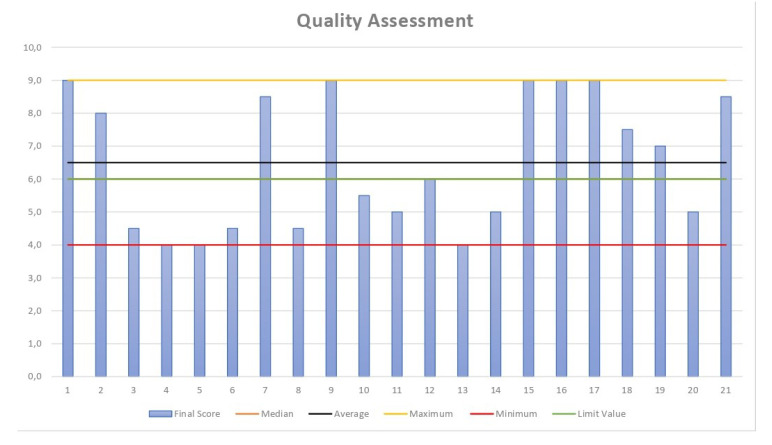
Distribution of quality data.

Figure 6 illustrates the distribution of quality data. The median score of the 21 articles included in the quality assessment stage was 6.0, and the mean was 6.5.

The selected articles are those in which the quality questions were applied (to assess) and from which the relevant data were extracted. The selected articles are shown in (Table 13). The articles were obtained at the beginning of the process (of planning and carrying out the review) once they had sufficient quality and affinity within the research questions and quality issues.

The selected articles are shown in Table 13.

In Table 14, all the results obtained with the data extraction process will be indicated. Answers to extraction questions DQ1, DQ2, DQ3, DQ4, DQ5, and DQ6 are presented.

It is important to highlight that the Scopus and Springer Link libraries were the databases in which the largest number of articles were found. The libraries wherein the least amount of articles were found were ACM and IEEE. The articles selected, in the inclusion stage, are mostly very recent, and most of them were published in 2021 and 2022.

### 3.2. Results Analysis

After applying the data extraction phase for all these articles, information is obtained on all the digital twins found. Table 15 lists all 11 digital twin results found, as well as their respective references. Digital twin applications were mostly related to the cultivation of a specific crop, the use of urban farming techniques, and conceptual reviews.

The use of terms related to ontology and multi-agents were often cited in many of the articles [41,55,59]. The terms related to IoT and big data were often cited in [7,18,46,50,56,57]. In addition, the terms related to artificial intelligence and intelligent systems were often cited in [7,18,41,46,53,56].

Table 15 presents the relevant information about each application of digital twins in agriculture, extracted from the last review step and based on the data extraction questions presented in Section 2.1.6. In the first column, each digital twin is related to the questions, and in the second column, each topic is associated with them.

The digital twins listed in Table 15 are described in Table 16, as some digital twins were mentioned in the articles without any detailed information. Table 16 presents the applications of digital twins, in which it was possible to obtain data through the selected works.

At the end of the systematic literature review and after the extraction of data from all accepted articles, the previously proposed research questions (Section 2.1.1) were answered. Furthermore, in Table 16 the results of the research questions are presented with a description of the applications of digital twins (taken from the data extraction phase).

At the end of the systematic literature review and after the extraction of data from all accepted articles, the research questions (Section 2.1.1) were answered. Furthermore, the answers are presented below.

RQ1: In the context of agriculture, what are the applications of digital twins?

Within the context of precision agriculture, they were categorised into different application sectors. The results found are associated with the cultivation of crops, such as wheat and “ginseng berry”. For example, the DT service for rice cultivation was developed, which allows the planning and modelling of the rice cultivation process according to climatic conditions [41]. A conceptual model of the digital twin is proposed. The proposed DT model is implemented in the laboratory environment for the cultivation of “ginseng berries” [46]. It describes a prototype system for a DT smart plant and discusses the main results and perspectives for the development of the digital twin system in wheat cultivation. [55]. The WebGIS framework is presented as an organising principle that connects local data generators and so-called site-specific smart farms to a regional and global networked view of agriculture that can support both the agricultural industry and policymakers in government [59].

The results found are used as important tools to boost the state of the art in a specific field. This review describes the state-of-the-art digital twin concepts, along with different digital technologies and techniques in agricultural contexts [7]. It is a review of emerging and disruptive technologies for urban agriculture [50]. One study is a review of research work carried out on the application of DT in smart agriculture (predictive analysis in hydroponics) [56].

Results related to different applications were found. That is, the use of irrigation presents the primary development of a digital twin for smart agriculture using IoT to control an irrigation system based on farmer decisions and/or AI [18]. Associated with city-level agriculture, we evaluated the effectiveness of a model-based digital twin approach and a machine learning approach to perform predictive decision analysis to predict urban agriculture production (a scalable aquaponics facility) [53]. Results were found about controlled-environment agriculture, and the digital twin of the underground farm faithfully represented the reality of the environment through real-time data streams, making it a useful representation for a farm operator [54]. Finally, concerning sustainable precision agriculture and the environment, the WebGIS framework is an organising principle that connects local data generators and so-called specific smart farms to a regional and global network view of agriculture. This will help to integrate databases located on networks into a system to achieve the necessary SPAE management and connect different fields [57].

RQ2: In these digital twins, what are the predictive control techniques and control actions employed?

After analysing the data obtained, the responses were organised in different domains and correlated with the control actions found. The results found are associated with multi-agent technologies: the monitoring and control of plant growth and development in fields using the digital twin. To support the decision-making process and management of agricultural production, ontologies and multi-agents must be used [41]. Data from the physical world, such as climate, fertiliser, and soil type, as well as information from developed models that simulate soil and crop behaviour, were considered as input data for the digital twin. The digital twin concept also consisted of a soil agent (includes hydrological models and soil data), a crop agent (includes crop models and evaporation data), and a field avatar, which is a digital representation of the field, such as geological models and climate data [7]. Cyber–physical systems (CPS) are a new type of system, integrating computing, communication, and control components, including sensors, actuators, and network connectors. Modern precision agriculture technologies with daily controlled plant cultivation can significantly improve product quality and agricultural production efficiency. This approach hypothesizes that agronomists and other experts in farm management can model a self-organising process of the entities to be implemented using multi-agent technology. Furthermore, it improves the quality and efficiency of agricultural management decisions [59].The results found are related to predictive control, the architecture of necessary for implementation at a farm level, data acquisition, and a pre-processing step. Thus, advanced analysis and modelling predictors can be implemented to allow for global coordination and optimisation. A use-case study is operational management. This case is supported by using real-time data from sensors to perform adaptive control and tactical management [53]. Within the limitations of the data, crucial variables for tracking and prediction are identified. The data model was introduced, which is essentially a predictive model that predicts extreme temperatures and provides feedback on operational changes that can reduce energy usage and control the farm environment more efficiently. We conclude with a discussion of the development of this digital twin. The five environmental variables that are continuously monitored are temperature, relative humidity, CO_2_ concentration, airspeed, and light levels [54]. By using DT, the relationship between nutrient solution temperature and meteorological factors can be found, leading to the development of a predictive model for nutrient solution temperatures. The various methods suggested by hydroponics producers for controlling the temperature of the nutrient solution include the use of centrifugal fans, squirrel-cage fans, or even air conditioners [56]. The main results found are related to the management of farms, and a monitoring system was developed for a farm that could collect and analyse information. On the farm, there are various devices and systems deployed, such as soil probes, weather stations, irrigation systems, seeders, harvesters, etc., [18]. The proposed digital twin model was implemented at the laboratory and field levels. According to weather conditions, electrical power is automatically supported or interrupted. The digital twin plays a role in farm management to enable self-monitoring and control of systems. The system is fully controlled and monitored based on digital twins and IoT [46]. With these architectural advances in farm management, the modern farm can resemble the notion of “digital twins”, which is the confluence of IoT, AI, and big data. Digital twins mean that agricultural operations no longer require physical proximity, which allows for the remote monitoring, control, and coordination of agricultural operations [57]. The results found are associated with other forms of mitigating actions. This, coupled with the coordination of agricultural operators, means that the digital twin uses simulation and AI to mirror system properties and behaviours in real time, incorporating all the statuses and information of the physical system. With digital twins, agricultural operators do not need to be physically at the agricultural site to monitor, control, coordinate, and run agricultural operations. Any changes to the physical system can be reflected by its digital counterpart. The digital twin will support decision making without the need to create prototypes [50]. Finally, associated with monitoring the state of nutrients, if the DT is synchronised with a plant (physical), it can adequately reflect its state, such as through regular inspections. They can be used by agronomists to develop and make management decisions in carrying out agro-technical measures based on planning and modelling possible problem situations and finding ways to solve them [55].

RQ3: Is this minimisation of the impact caused on the soil based on reducing the application of chemical and mechanical actions?

Within the context of precision agriculture, they were categorised into different actions related to the reduction in impacts on the soil. The results found are related to resource management, such as the implementation of DT in decision making on the efficiency and sustainability of agriculture under global climate change. The platform determines the control of agro-technical conditions, including the determination of the volumes, concentrations, and application rates of fertilisers and phytopharmaceutical products (plant-protection products) as a function of climate [41]. The use of digital twins makes it possible to determine the parameters that affect the behaviours of the farm and the final production and consumption of resources. This is a key feature that allows farmers to make better decisions and lessen the environmental impacts on resources [18]. Virtual models of farm operating parameters can guide agricultural operators in making decisions, thus maximising yields and minimising energy and water use. Digital twins can act as safe operating boundary monitoring systems [50]. Monitoring and evaluating soil quality can reduce the potential use of chemical fertiliser and pesticide dosages, improve groundwater, and protect the environment and human health. This also supports setting the plant density more efficiently. Soil monitoring sensors, such as sensors for moisture, temperature, organic matter, and soil pollutants, can provide soil moisture information that can be used to assess irrigation efficiency in the agricultural field [7]. The digital plant twin will be created for each field to mirror the actual growth and development of the plant, representing the most anticipated version of the plant development plan, updated daily with data from the weather server, sensors in fields, and observations from agronomists. The main idea of the proposed approach is to consider crop cultivation as a complex adaptive system with collective decisions [59].

The results found are related to soil-less cultivation, namely hydroponics and the process of developing a digital twin of a unique underground hydroponic farm. The key to the continued operational success of this farm and similar ventures is finding ways to minimise energy use and maximise crop growth while maintaining optimal growing conditions in which indoor environments are controlled [54]. Hydroponics is one of the popular soil-less ways to grow plants indoors, which reduces fertiliser usage and provides more protection from pests and adverse weather conditions. It aims to study the environmental performance of various nutrient-recovery methods [56].

Results were found related to various actions to reduce impacts on the ground. That is, in urban agriculture, food security, resilience to climate disturbances, environmental sustainability, and positive economic and social outcomes have been identified. By predicting the effect of agricultural policy decisions on social and economic variables [53], SPAE will contribute to increasing sustainability, reducing nutrient transport [57]. Finally, it investigates actions that are related to monitoring the state of nutrients

The project is operated inside the building where the plant is growing in water with nourishment and without any fertiliser, soil, or sunlight. Several sensors are connected to the plant’s control module, which monitors the plant’s nutrient and growth status and external weather conditions as well. [46]. Knowledge about the microstates of plant development should help to more accurately model and predict plant growth [55].

### 3.3. Discussion

In the present work, a systematic literature review was carried out. This work intended to survey the scientific publications, produced since 2016, that had developed or worked with a soil’s digital twins in the context of smart farming and precision agriculture. Particularly, the interest lies in the use of the soil’s digital twin in the context of a closed-loop predictive control, where this digital twin, fed with real-time data, can be used to carry out short- and long-term forecasts.

Following the methodology used in this review, a total of 158 articles were found and extracted from five different electronic databases: Springer Link, Association for Computing Machinery, ISI Web of Science, Institute of Electrical and Electronics Engineers, and Scopus. While performing the search, it was possible to build a deeper insight regarding the overall amplitude of this particular researched field and its current state of the art. Moreover, it is important to highlight that the Scopus and Springer Link libraries were the databases in which the largest number of articles were found and classified. Both ACM and IEEE were the libraries in which the least amount of articles was found. In particular, the least-ranked articles were extracted from the ACM Digital Library. It is worth noting that the articles selected during the inclusion stage were mostly recent, since they were published between 2019 and 2022.

After applying the inclusion and exclusion criteria, the quality assessment stage identified 11 relevant articles to be included in the final report review. Among them, the most often cited investigated the cultivation of a specific culture, water management platforms, and urban agriculture techniques, and performed systematic reviews. Moreover, some keywords were found in most articles. For example, the use of ontology- and multi-agent-related terms were often cited in many of the articles [41,55,59]. The terms related to IoT and big data were often cited in most articles [7,18,46,50,56,57]. The words related to artificial intelligence and intelligent systems were also present in the large majority of papers [7,18,41,46,53,56].

In view of this, it can be concluded that the amount of papers associated with the use or development of soil’s digital twin is being gradually increasing. Indeed, after the systematic review carried out in this work, it is possible to conclude that there is a growing trend toward the use of digital twins within various domains and with different goals. However, no studies address the precise problem of soil quality control in general and within a predictive control framework in particular.

## 4. Conclusions

In the present century, agricultural producers face challenges to preserve water and soil while achieving food safety and promoting sustainability. The latter is being tackled by resorting to more modern farm management and cultivation approaches that jointly use ecological technologies (plant-cover plants that are beneficial to other plants) and precision agriculture. Precision agriculture is a broad concept that includes the use of sensors and information technology to improve the many processes that occur in agriculture, such as irrigation and fertilisation. Sustainability and soil-quality management are strongly dependent on the surgical deployment of fertilisers and/or pesticides, which must be accompanied by an increase in crop yield [57].

Taking into consideration the research results that document the application of digital twin paradigms in the industry, it is expected that deploying equivalent approaches in agriculture will lead to an increase in productivity while keeping a leaner trend regarding the use of energy, water, fertilisers, and pesticides. In this framework, digital twins not only will help farmers to increase crop yields but also reduce production costs and allow them to grow crops with better nutritional value [60].

Digital twins are still in the process of definition and it seems that the advantages of their use are especially pronounced when standard assets management and control systems are of limited utility [54,61]. Examples of digital twins that successfully and perfectly match these elements in a complex operational environment are rare or even non-existent.

After a thorough and careful search of several databases, a set of relevant articles aligned with the the use of digital twins in agriculture were found to be available in the scientific literature. Some of those publications are focused on studies and exploratory cases. Some of the points-of-view presented by the authors, such as the use of digital twins to organise, monitor, manage, and maximise agricultural procedures, require further explanation and validation.

According to the literature, the slow penetration of digital twins on agricultural processes occurs due to several reasons, such as low amounts of technical resources, the lack of ease of communication on remote farms, a shortage of economical funding, an unpredictable environment and continuous changes in climate, soil quality, producer resistance in sharing their agricultural information, and the low-level technical qualifications of agricultural growers [56]. Nevertheless, over the past decade, there are records on the use of digital twin technology employed in the context of smart farming [46]. Usually, the aim of such an approach is to promote the separation between the physical flows and the planning and control. In particular, providing the grower with the ability to remotely manage all the systems in real time by using virtual information [18].

Based on the results obtained from this systematic review, it is possible to conclude that there are no relevant scientific papers published on the use of digital twins for soil-quality management. Moreover, no papers were found regarding the use of such a paradigm in the context of model predictive control for the closed-loop regulation of fertilisers or pesticides.

## Figures and Tables

**Table 1:** Key phrases used in the database query. Questions.

Search Questions		
**RQ1**	**RQ2**	**RQ3**
In the context of agriculture, what were the applications of digital twins?	In these digital twins, what were the control techniques or actions employed?	Was there a concern or action to minimise the impact caused on the soil based on reducing the application of chemical and mechanical actions?

**Table 2:** The PICOC method.

PICOC Method				
**Population**	**Intervention**	**Comparison**	**Result**	**Context**
Digital twins	Soil digital twins and predictive controller; machine learning	Compare with existing digital twins	Optimisation of soil quality and reduction in the number of pesticides and fertilisers applied to the soil	Precision agriculture

**Table 3:** Selection of keywords and synonyms.

Definition of Keywords		
**Keywords**	**Synonym**	**Related to**
Digital twin	-	Population
Soil	land, field	Context
Agriculture	crop, farm	Context
Field management	-	Intervention
Soil quality	-	Result

**Table 4:** Inclusion criteria.

Inclusion Criteria	
IC1	Paper is accessible
IC2	The work was published after 2016
IC3	The article belongs to the thematic area, namely precision agriculture
IC4	The article includes one or more keywords, has an adequate structure, and proposes some kind of implementation initiative
IC5	The paper is written in English

**Table 5:** Exclusion criteria.

Exclusion Criteria	
EC1	Paper is not accessible
EC2	The work was published before 2016
EC3	The article does not belong to the thematic area, namely precision agriculture
EC4	The article belongs to the thematic area, but only to redefine general concepts
EC5	The article does not include the keywords: “digital twins”
EC6	The article includes some of the keywords, but only to redefine general concepts
EC7	The paper is not written in English

**Table 6:** Quality Questions.

Quality Questions	
QQ1	Was the article based on research and not on expert opinion?
QQ2	Does the article have a clear research objective?
QQ3	Does the article discuss the results of the work?
QQ4	Was the development context of the article an agricultural environment?
QQ5	Was the application of one or more digital twins discussed in the article?
QQ6	Were the applications of digital twins sufficiently characterized?
QQ7	Were the challenges and activities of applying digital twins discussed in the article?
QQ8	Were the contributions and benefits of soil digital twin applications discussed in the article?
QQ9	Does the article answer at least one of the research questions?

**Table 7:** Data extraction questions.

Data Extraction Questions	
DQ1	What were the main applications of digital twins?
DQ2	What were the main objectives?
DQ3	What were the main challenges/obstacles faced?
DQ4	What were the main technologies used?
DQ5	What were the main contributions found?
DQ6	Did the article discuss the applications of soil digital twins?

**Table 8:** Specific search phrases for each digital library.

Digital Library	Specific Search String
Springer Link	(“digital twin* ”) AND (agri* OR crop* OR farm*) AND (soil OR land OR field OR “field* management*“ OR “soil quality*”)
ACM	[All: “digital twin”] AND [[All: agri*] OR [All: crop*] OR [All: farm*]] AND [All: “soil”] OR [All: “land”] OR [All: “field ”] OR [All: “field management*”] OR [All: “soil quality *”]]
WoS	(“digital twin*”) AND (agri* OR crop* OR farm*) AND (soil OR land OR field OR “field* management*” OR “soil quality*”).
IEEE	(“digital twin*”) AND (agri* OR crop* OR farm*) AND (soil OR land OR field OR “field* management*” OR “soil quality*”)
Scopus	ALL ( (“digital twin*” ) AND (agri* OR crop* OR farm* ) AND ( soil OR land OR field OR “field management*” OR “soil quality*” ) ) AND (LIMIT-TO ( DOCTYPE, “ar” ) AND (LIMIT-TO ( SUBJAREA, “ENGI” ) AND ( LIMIT-TO ( EXACTKEYWORD, “digital twin” ) )

**Table 9:** Percentage of articles by libraries.

Percentage of Articles by Libraries		
**Libraries**	**Number of Articles Extracted**	**Percentage of Selected Articles**
SL	56	35
ACM	09	6
WoS	31	20
IEEE	11	7
Scopus	51	32
Total	158	100

**Table 10:** Quality Assessment Result.

Quality Assessment Result											
**Reference**	**Number**	**QQ1**	**QQ2**	**QQ3**	**QQ4**	**QQ5**	**QQ6**	**QQ7**	**QQ8**	**QQ9**	**Final Score**
[41]	1	Y	Y	Y	Y	Y	Y	Y	Y	Y	9.0
[18]	2	Y	Y	Y	Y	Y	P	P	Y	Y	8.0
[42]	3	Y	Y	N	Y	Y	N	N	N	P	4.5
[43]	4	Y	Y	Y	N	Y	N	N	N	N	4.0
[44]	5	Y	Y	Y	Y	N	N	N	N	N	4.0
[45]	6	Y	Y	Y	Y	N	N	N	N	P	4.5
[46]	7	Y	Y	Y	Y	Y	Y	P	Y	Y	8.5
[47]	8	Y	Y	Y	Y	N	N	N	N	P	4.5
[7]	9	Y	Y	Y	Y	Y	Y	Y	Y	Y	9.0
[48]	10	Y	Y	Y	Y	N	N	P	P	P	5.5
[49]	11	Y	Y	Y	Y	N	N	N	N	Y	5.0
[50]	12	Y	Y	Y	Y	N	N	P	P	Y	6.0
[51]	13	Y	Y	Y	Y	N	N	N	N	N	4.0
[52]	14	Y	Y	P	Y	Y	P	N	N	P	5.0
[53]	15	Y	Y	Y	Y	Y	Y	Y	Y	Y	9.0
[54]	16	Y	Y	Y	Y	Y	Y	Y	Y	Y	9.0
[55]	17	Y	Y	Y	Y	Y	Y	Y	Y	Y	9.0
[56]	18	Y	Y	P	Y	Y	P	P	Y	Y	7.5
[57]	19	Y	Y	Y	Y	P	P	P	P	Y	7.0
[58]	20	Y	Y	P	Y	Y	P	N	N	P	5.0
[59]	21	Y	Y	Y	Y	Y	P	Y	Y	Y	8.5

Subtitle: Y—YES: 1.0 (highest score if you answer the questions completely); P—PARTIALLY: 0.5 (average score if you partially answer the question); N—NO: 0.0 (lowest score if you do not answer the question).

**Table 11:** Articles Selected and Accepted by year.

Articles Selected and Accepted by Year		
**Year**	**Selected**	**Accepted**
2017	2	-
2018	1	-
2019	22	4
2020	29	4
2021	67	10
2022	33	3
Total	158	21

**Table 12:** Statistical Calculations.

Statistical Calculations				
**Median**	**Average**	**Maximum**	**Minimum**	**Limit Value**
6.0	6.5	9.0	4.0	6.0

**Table 13:** Selected articles.

Selected Articles			
**Identification**	**Reference**	**Authors**	**Final Score**
Case study 01	[41]	P. Skobelev	9.0
Case study 02	[18]	R. G. Alves	8.0
Case study 03	[46]	Y. Sung	8.5
Case study 04	[7]	Nasirahmadi	9.0
Case study 05	[50]	A. K. Ng and Mahkeswaran	6.0
Case study 06	[53]	Ghandar	9.0
Case study 07	[54]	Jans-Singh	9.0
Case study 08	[55]	P. Skobelev	9.0
Case study 09	[56]	T. Sreedevi	7.5
Case study 10	[57]	J.A. Delgado	7.0
Case study 11	[59]	V. Laryukhin	8.5

**Table 14:** Data Extractions Results.

Data Extractions Results	
Case 01	[41]
DQ1	Rice Cultivation
DQ2	Determining plant growth patterns and improving crop yields
DQ3	Global climate change puts agricultural production at risk
DQ4	Digital platform for intelligent services, an intelligent system based on ontology and multi-agent technology
DQ5	Increase in business efficiency, increase in income, decrease in the complexity of business management, high transparency and traceability of operations, reduction in errors due to the negative human factor, and business growth with reduction in administrative costs
DQ6	Y
Case 02	[18]
DQ1	Water management platform and soil probe
DQ2	Improvement in crop water management and understanding of the best state of farms in terms of use of resources and equipment
DQ3	Factors such as climate change and the expansion of the world population have become a global challenge for the availability of fresh water and insufficient irrigation, causing a reduction in crop productivity
DQ4	IoT, big data, artificial intelligence (AI), process management, IoT Fiware agent, MYSQL, Grafana
DQ5	With the final system in place, it will be possible to understand the consumption of resources on the farms and the impact on crop yields. This will enable sustainable development and increase food security for the global population
DQ6	Y
Case 03	[46]
DQ1	Cultivation of ginseng berry
DQ2	Allowed to decouple the physical flow from cyber control system and implement a digital twin conceptual model for smart farms
DQ3	In the IoT vision, a high level of interoperability needs to be achieved in terms of communication, as well as in service and even in the levels of knowledge on different established platforms
DQ4	Monitoring, sensing, smart farm technology, and smart big data analytics equipment
DQ5	The digital twin smart farm model was suggested, and the implementation was presented at the laboratory level and at the field level. The ginseng plant was adopted and tested in the proposed system
DQ6	Y
Case 04	[7]
DQ1	Review of digital twin concepts
DQ2	Provided an overview of digital twins in the ground. Data recording and modelling, including artificial intelligence, big data, simulation, analysis, forecasting, and communication aspects are discussed
DQ3	One of the main global challenges has been how to guarantee food security for the world’s growing population, ensuring long-term sustainable development
DQ4	Information and communication (ICT), Internet of Things (IoT), big data analysis and interpretation techniques, machine learning, and artificial intelligence
DQ5	Through real-time continuous monitoring of the physical world (the farm), it was possible to update the state of the virtual world. Data-driven approaches increased decision-making capabilities on the farm, improving crop performance, reducing losses, and therefore benefiting the crop
DQ6	Y
Case 05	[50]
DQ1	Review of urban agriculture techniques
DQ2	A comparison between different technologies (including digital twins) was presented. A list of their applications, advantages, and disadvantages was discussed
DQ3	In agriculture, environmentally unsustainable practices were adopted, which can lead to deforestation
DQ4	Fusion of IoT and AI, known as Artificial Intelligence of Things (AIoT), was used, along with the latest eco-friendly fifth-generation wireless technology (5G), green 5G-AIoT
DQ5	Provided a reliable and energy-efficient network of interconnected smart devices capable of self-monitoring and self-healing
DQ6	Y
Case 06	[53]
DQ1	Urban agriculture: aquaponics
DQ2	Scalable aquaponics installation
DQ3	Research on aquaponics in low- and middle-income countries often focused on food security, as in the Gaza Strip, an arid and dense urban area in prolonged crisis
DQ4	Digital twin system and machine learning gateway
DQ5	Several potential benefits have been demonstrated, for example in the reduction in waste and logistical costs. The growing acceptance of urban agriculture can reduce the production load and generate benefits of greater food security and sustainability
DQ6	Y
Case 07	[54]
DQ1	Controlled-environment agriculture
DQ2	The digital twin presented here proposed a framework for integrated urban farms to collect data and use it in a meaningful way
DQ3	In implementing digital twins, some challenges need to be overcome (in data creation, data analysis, and data modelling). If the farms were to test different lighting regimes at different times of day and different ventilation rates for prolonged periods in different weather conditions, more could be learned about the response of the farm environment to the controls
DQ4	Used a wireless sensor network (WSN) that sends real-time data to a server. The WSN is composed of 25 sensors, monitoring a total of 89 variables, which transmit data to 8 Raspberry Pi registers. These registrars, in turn, transferred the data to servers in the Engineering Department at the University of Cambridge (server) over WiFi. Loggers also stored data on SD cards when wireless service dropped. Microsoft Azure database
DQ5	Farm grew 12 × more per unit area than traditional greenhouse farming in the UK. The farm also consumed 4 × more energy per unit area
DQ6	Y
Case 08	[55]
DQ1	Wheat cultivation
DQ2	The article proposed a method to estimate the duration of plant development stages and yield based on expert knowledge. A method was presented to calculate the yield forecast, as well as the start and end dates of each stage of plant development within the tube during its normal development and in case of critical situations. Described the structure and functions of a DT smart plant, which was built on a module for the multi-agent planning of plant development stages and integrated with the external weather forecast and fact services. A brief description of the smart plant DT system prototype in Java was provided
DQ3	In agricultural production, it has generally been very difficult to plan the work, even with precision in the composition and order of operations, which is due to the great lack of knowledge about plant life, characterised by high complexity, uncertainty, and dynamics, mainly caused by climate change. When using machine learning models, a test selection is required, which must be achieved under certain conditions unchanged
DQ4	Digital twin
DQ5	New principles for construction and implementation of the digital twin plant; a knowledge base for the development of wheat stages; the structure and functions of the DT intelligent system (using wheat as an example), open to supply with other crops; a prototype smart plant DT system in Java; and a protocol tested on model data to prove its practical applicability for greater compliance, building the knowledge base, making calculations and decisions
DQ6	Y
Case 09	[56]
DQ1	Review on the application of digital twin (DT)
DQ2	The application of the Hydroponic method, in which different ways were described in which DTs can contribute
DQ3	Digital twins (DT) have a huge margin of success in the field of sustainable agriculture. However, the number of works carried out in this field was relatively less compared with other domains. There is a great need to adopt more efficient and sustainable production methods. To support this, a detailed review of next-gen DT apps in smart agriculture was performed in this article. It found that challenges, such as natural disasters, soil erosion, climate change, urbanisation, and epidemics, are making soil-less farming methods increasingly popular compared with soil cultivation
DQ4	Technologies, such as big data analysis, robotics, Internet of Things, and artificial intelligence
DQ5	Smart farming methods have been invented to meet the growing demand for global food production. The digital twin has been identified as an excellent candidate for making these farming methods more efficient. DT can involve different phases of the hydroponic agriculture lifecycle
DQ6	Y
Case 10	[57]
DQ1	Sustainable precision agriculture and environment (SPAE)
DQ2	Developed a digital twin that can allow the simulation of new ideas that can be tested virtually to determine environmental impacts before real-world implementation
DQ3	The greatest challenge of the century is achieving food security. Agricultural systems face challenges, such as climate change, depletion of water resources, potential erosion and loss of productivity due to the occurrence of extreme weather events, low adherence to decision support tools, poor communication infrastructure, siloed data management, and immature AI analytics applications
DQ4	Big data, digital agriculture, WebGIS framework, automation, IS, IoT, DRONES, roos, digital twins
DQ5	Precision agriculture emerged as a way to improve margins by managing input costs and at the same time improving yields. Contributed to increased income and profits, greater adaptation to climate change, and greater sustainability (off-field and across the watershed)
DQ6	Y
Case 11	[59]
DQ1	Wheat cultivation
DQ2	A conceptual model of the digital twin of the plant-based on ontology was proposed, which corresponds to the macro stages of plant development with the possibility of recalculating its parameters
DQ3	In an agricultural context, it becomes difficult to plan operations, with high adaptability required. The problem becomes more complex, with the increase in climatic variations, little knowledge about plant development factors, and the high uncertainty of the cultivation
DQ4	Multi-agents and ontologies
DQ5	Under conditions of global climate change, it is possible to create a system that will help to accumulate invaluable agronomic experience and take into account the new realities of agricultural production. Continuous monitoring and control of plant development phases will allow for the timely detection of deviations from the norm and the development of immediate recommendations for measures to reduce risks and damage to crops
DQ6	Y

**Table 15:** All digital twins and their respective references.

Digital Twins		
**Reference**	**Applications**	**Financing**
[41]	Rice Cultivation	It has no information
[18]	Water management platform and soil probe	It has no information
[46]	Cultivation of “ginseng berry”	Support from the Basic Scientific Research Program through the National Research Foundation of Korea (NRF) funded by the Ministry of Education
[7]	Review of state-of-the-art digital twins concepts	Did not receive external funding
[50]	Review of urban agriculture techniques	It has no information
[53]	Urban farming: aquaponics (growing plants and fish together)	It has no information
[54]	Controlled-environment agriculture	Support from the Engineering and Physical Sciences Research Council at the University of Warwick. Furthermore, funding also by AI for Science and Government (ASG), the UKRI Strategic Priorities Fund awarded to the Alan Turing Institute, and the Lloyd’s Register Foundation program in Data-Centred Engineering.
[55]	Wheat cultivation	Supported by the Ministry of Education and Science of the Russian Federation
[56]	Review on digital twin applications in smart agriculture	It has no information
[57]	Sustainable precision agriculture and environment (SPAE)	It has no information
[59]	Wheat cultivation	Support from the Ministry of Education and Science of the Russian Federation at the State Technical University of Samara.

**Table 16:** Research questions results.

Research Questions Results	
Case study 01	[41]
RQ1	It is developed as a stand-alone service and can be integrated with any existing digital agriculture platform. A pilot integration with the cyber–physical system for agriculture needs
RQ2	Fast, flexible, and efficient planning of agro-technological operations, as well as the subsequent control of the implementation of selected cultivation technologies. Monitoring and control of plant growth and development in fields using the digital twin
RQ3	The system performs adaptive scheduling of resources, such as fertilisers, protection agents, vehicles, personnel, and finances. Implementing DT in proper service decision making compared with pilot farming experiments makes businesses smarter, more flexible, and cost-effective, providing better plant cultivation productivity and agriculture sustainability to combat global climate change. The idea of accurate agricultural mail is that field processing is performed based on the actual state of crops at a given time and place. These needs can be determined by several modern information applications, namely remote sensing. At the same time, the treatment means are differentiated in several areas of the field, providing the best efficiency with the minimum environmental impact and reducing the amount of waste used
Case study 02	[18]
RQ1	The Sensing Change project developed a soil probe, while the SWAMP project is currently developing an Internet of Things platform for water management on farms. This article leverages the technologies developed by these projects by building an initial digital environment to create a cyber–physical system (CPS) so that farmers can better understand the state of their farms in terms of resource and equipment usage. The system can collect data from the land probe and display its information on a dashboard that allows for the deployment of more land probes and other monitoring and control devices to create a fully operational digital twin. Presents the primary development of a digital twin for smart agriculture using IoT to control an irrigation system based on farmer decisions and/or AI
RQ2	Project consists of a monitoring station, a smartphone app, and a cloud system. A monitoring system was developed for a farm that could collect and analyse information. Proposed system: On the farm, there are several devices and systems deployed, such as soil probes, weather stations, irrigation systems, seeders, harvesters, etc. These devices and equipment are connected to the cloud through a gateway that sends information to an IoT Agent (a service that translates various communication protocols into the one used in the cloud). However, to fully develop a smart digital farm, all environments must be developed using an integrative approach. Data collected and analysed in the cloud as viewed in the digital environment must be entered into the physical system via the cloud or by connecting programmable logic controllers (PLCs) in the irrigation system, equipment, and machines
RQ3	By using the digital twin model and IoT technology, farmers can connect different assets and systems to gain greater insight into the different aspects and parameters that affect farm behaviour and final production and resource consumption. This is a key feature that allows farmers to make better decisions and reduce environmental impacts to water, land, and soil resources. This research indicates that the system design and cloud implementation are working and can be used in the implementation of the next steps, which are the development of AI algorithms and other digital contexts.
Case study 03	[46]
RQ1	The main contributions and meanings of this study are to suggest the digital twin smart farm architecture and to implement the concept in the laboratory environment for a practical point of view. This shows how smart farm architecture can be realised based on digital twin technology. The concept is also applied in the smart farm environment itself, which shows the possibility of a commercial success story. Prescriptive DT: An intelligent digital object that aggregates intelligence to recommend corrective and preventive actions on real-life objects, usually based on optimisation algorithms and specialised heuristics, using predictive analytics. This article sets out to explore the recent trend of digital twin modelling prevalent in the smart farm context. After a literature review, the conceptual framework of the DT is proposed
RQ2	Digital twins have been adopted in smart agriculture across wide areas in the last decade. Digital twins can play a central role in farm management, which allows for decoupling the physical flow from the cyber control system. In a smart farm environment, farmers can be free from soil or farmland. Instead, they can control and monitor the status of farming in the build room while using the monitor. This transforms agricultural activities into different dimensions compared with those used in the past. Several sensors are connected to the plant’s control module, which monitors the nutrient status and growth of the plant. External weather conditions are also monitored by sensors installed on the laboratory ceiling. According to weather conditions, electrical power is automatically supported or interrupted. The self-monitoring and control system plays the role of the digital twin in the DT system. We designed the LED and LD (laser diode) wavelength-controlled vegetable growing system that optimises the elements of plant growth. It is fully controlled and monitored based on digital twins and IoT. The actual growing system is designed using a laboratory-grade container
RQ3	All field crops need soil, light (sun), temperature, air, water, and nutrition to grow. Soil gives plants stability; it also stores water and nutrients that plants can absorb through their roots. Light (sunlight) provides the energy needed for plant growth. Air allows plants to “breathe”. Water provides moisture and nutrition. The practical architecture of DT is explained. The smart farm is free of agriculture essentials, such as soil, sunlight, air, water, and fertilisers. It is designed and operated inside the building where the plant is growing in the water with nourishment and without any fertiliser, soil, and sunlight
Case study 04	[7]
RQ1	This review describes the state-of-the-art digital twin concepts, along with different digital technologies and techniques in agricultural contexts. It presents an overview of digital twins in soil, irrigation, robotics, agricultural machinery, and post-harvest food processing in the agricultural field. Data recording and modelling, including artificial intelligence, big data, simulation, analysis, and prediction, as well as the communication aspects of digital twins in agriculture are discussed. Digital twin systems can support farmers as a next-generation digitalisation paradigm, continuously monitoring the physical world (farm) in real time and updating the state of the virtual world
RQ2	Data from the physical world (agricultural area), such as climate, fertiliser, and soil type, as well as information from developed models that simulate soil and crop behaviour, were considered as input data for the digital twin. The digital twin concept also consisted of a soil agent (including hydrological models and soil data), a crop agent (including crop models and evaporation data), and a field avatar, which is a digital representation of the field, such as geological models and climate data. Exchanging information from soil as a physical system to a virtual system using IoT, cloud, fog, and edge computing technologies in digital twins can allow us to assess the state of soil and irrigation systems. In particular, an edge computing technique that saves and executes data processing next to soil and irrigation monitoring devices can improve performance and overcome the problems of cloud-based systems in digital twin concepts. In addition, it could offer different irrigation recommendations based on crop needs that have not yet been resolved by researchers
RQ3	Monitoring and evaluating soil quality to sustain plant productivity is the basis of land-use strategies on agricultural farms. The health and productivity of crops depend on the quality and property of the soil. More detailed information about soil cultivation can reduce the potential use of chemical fertiliser and pesticide dosages, improve groundwater, and protect the environment and human health. This also allows you to define the plant density more efficiently. Digital technologies are helping scientists better understand and study the soil. Soil monitoring sensors, such as sensors for humidity, temperature, organic matter, and soil pollutants, are playing a critical role in digital agriculture
Case study 05	[50]
RQ1	In this article, several emerging and disruptive technologies for urban agriculture are reviewed and evaluated. Based on the literature from 2015 to 2021, IoT, automation, and AI are the top three technology innovations that are widely implemented and documented. In contrast, genetic modification, AM, and nanotechnology are relatively new and are in the early stages of adoption
RQ2	A digital twin is a virtual representation of a physical system. It uses simulation and AI to mirror system properties and behaviours in real time, incorporating all physical system statuses and information. Any changes to the physical system may be reflected by its digital counterpart. As such, a digital twin can illustrate how a physical system will react in different design alternatives and situations, supporting decision making without the need to create prototypes. With digital twins, farm operators do not need to be physically on the farm site to monitor, control, coordinate, and run farm operations. Simulating layers of vertical beds in different configurations optimises building resources. Virtual models of farm operating parameters (e.g., energy and water consumption) can guide agricultural operators in making decisions, thus maximising yields and minimising energy and water use. In addition to current data, historical data can be used to predict system behaviours. Thus, digital twins can act as early warning systems when the predicted environment goes outside safe operational limits. However, implementing digital twins for agriculture is complicated and demanding. Most agricultural variables are associated with living organisms and are difficult to accurately model and simulate because of their intricate behaviours. In addition, modelling and simulating the fertility of seeds, fertilisers, pesticides, and pollution is a challenge
RQ3	Virtual farm operation models can guide agricultural operators in making decisions, thus maximising yields and minimising energy and water usage. In addition, data is used to predict system histories. Thus, digital twins can act as early warning systems when the predicted environment exceeds safe operational limits. However, implementing digital twins for agriculture is complicated and demanding. Most agricultural variables are associated with living organisms and are defined from the model
Case study 06	[53]
RQ1	The article aims to describe the background and related works, namely to describe a planning and decision support system for coordinating multiple farms and planning agricultural initiatives at the city level, to describe the cyber–physical aquaponics system that was developed, and present the results and empirical analysis. In the results section, we evaluated the effectiveness of a model-based digital twin approach and a machine learning approach to perform predictive decision analysis to predict urban agriculture production (a scalable "aquaponics" facility). Were also evaluated the ability of a modelling framework to generate meaningful insights into urban agriculture system design as a step towards a decision support system that uses an online simulation.
RQ2	The system architecture required to implement the system from the level of individual farms, data acquisition, and through a pre-processing step to enable greater coordination at the cloud layer, where global optimisation and advanced analysis and modelling capabilities predictors can be implemented. A use-case study is operational management. This case is supported by using real-time data from sensors to perform adaptive control and tactical management. The decision support system would connect different stakeholders and allow them to coordinate activities through a gateway. These users include agricultural facilities, retailers, distributors, and consumers
RQ3	The benefits of urban agriculture have been identified in food security, resilience to climate disturbances, environmental sustainability, and positive economic and social outcomes. As an example, there is the possibility of reducing food waste by recycling food waste as fertiliser
Case study 07	[54]
RQ1	In this article, the digital twin of the underground farm faithfully represents the reality of the environment through real-time data streams, making it a useful representation for a farm operator. This includes three crucial elements: (a) Data Creation: An extensive and robust monitoring system that tracks observable environmental conditions at the underground farm. This is supported by data curation that ensures data quality and tractability; (b) Data analysis: Using observable data in conjunction with information reported by agricultural operators to identify key variables that influence the farm environment and therefore crop yields; (c) Data modelling: Investigating the most appropriate techniques to identify trends and critical changes, predict possible future operational scenarios, and provide feedback on the influence of recent events on the farm environment
RQ2	The structure of the article follows the representation of the digital twin. We first introduce the monitoring process and key data challenges of monitoring in a continuously operating environment. We present data analysis that includes: (a) the influence of the farm environment on crop growth, (b) the influence of operable controls on the environment, and (c) the influence of manual changes to operational controls. Within the limitations of the data, this exercise identifies the variables that are crucial to tracking and predicting. Next, we introduce the data model, which is essentially a predictive model that predicts extreme temperatures and provides feedback on operational changes that can reduce energy usage and control the farm environment more effectively. We conclude with a discussion of the development of this digital twin. The five environmental variables that are continuously monitored are temperature, relative humidity (RH), CO_2_ concentration, air velocity, and light levels. Some of them, such as temperature, are monitored by several sensors, linked to different data loggers. This differs from typical CEA predictive control models, where changes in control processes (heating, ventilation) are automatically regulated in response to short-term temperature predictions
RQ3	The process of developing a digital twin of a unique hydroponic underground farm in London, Growing Underground (GU). The key to the continued operational success of this farm and similar ventures is finding ways to minimise energy use while maximising crop growth and maintaining optimal growing conditions. As such, it belongs to the environmentally controlled agriculture class, where indoor environments are carefully controlled to maximise crop growth using artificial lighting and smart heating, ventilation, and air-conditioning systems
Case study 08	[55]
RQ1	Digital twin from the project to an intelligent cyber–physical system for precision agriculture management. The article discusses the constructive principles of the DT plant, as well as models, methods, and specific characteristics of its implementation, which is the basis of an intelligent cyber–physical system (ICPS) for precision agriculture management. It shows the main directions of digitalisation in agriculture associated with the beginning of the development of cyber–physical systems for precision agriculture. It presents the problem statement for creating a DT in the form of an intelligent decision support system using a detailed formalised representation of knowledge about each stage of plant development. Provides an overview of existing developments in the use of CPS and DTs. It describes the structure and functions of the IDT plant based on multi-agent technology and the ontological representation of knowledge. It proposes a new model to assess the yield and duration of plant development stages based on the range of change for the most important parameters of plant development at each stage. It examines the method for calculating the duration of the plant’s developmental stage depending on changes in temperature and crop yield. It describes a prototype system for a DT smart plant. It discusses the main results and perspectives for the development of the system with the IDT plant
RQ2	If the DT is synchronised with a real plant, i.e., it can properly reflect its state, e.g., through regular inspections of real plants by agronomists, it can be used by agronomists to develop and make decisions, such as whether to implement agro-technical measures based on planning, in addition to modelling possible problem situations and finding ways to solve them
RQ3	Knowledge about the microstates of plant development should help less qualified agronomists to more accurately model and predict plant growth and change plans for agro-technical measures on time, developing management actions in case of deviations from the actual development of the plant by the application of fertilisers. Regarding fertiliser application volumes, it is known that this is usually quite expensive, requiring loans that can only be returned after the harvest is sold
Case study 09	[56]
RQ1	A review of research works carried out on the application of DT in smart agriculture was presented. Performing predictive analysis in hydroponics using DT can solve many problems
RQ2	To improve the temperature prediction of the nutrient solution, the DT concept can be applied with the aid of meteorological data. By using DT, the relationship between nutrient solution temperature and meteorological factors can be found leading to the development of a predictive model for nutrient solution temperature. The various methods suggested by hydroponics producers to control the temperature of the nutrient solution are the use of centrifugal or squirrel-cage fans or even air conditioners. Using DTs, farmers can estimate the performance of such cooling devices when installed on a hydroponics farm without actually installing actual devices. It allows farmers to create an efficient initial design of their farm and evaluate the performance caused by adding new features such as fans and heaters
RQ3	Hydroponics are one of the popular ways of growing soil-less plants indoors, reducing fertiliser usage and providing more protection from pests and adverse weather conditions. Hydroponics challenges also include the need for capital investment and experience in operational control systems. Reducing the use of fertilisers used in hydroponics and the environmental performance of various nutrient-recovery methods are discussed
Case study 10	[57]
RQ1	The growing impact of climate change, the next revolution in precision agriculture and agriculture in general, will be driven by Sustainable Precision Agriculture and Environment (SPAE, similar to the 7 Rs ). This transitions from a site-specific management focus to a global sustainability notion. In this transition, it presents WebGIS as a principle that connects local data systems and as site-specific smart grid generators to an agricultural industry view. The increasing use of artificial intelligence (AI), the Internet of Necessary Things, drones, and big data, which will serve as the global basis for the “digital twin”, will contribute to the development of conservation practices, site-specific management that ensure the conservation, and general sustainability
RQ2	Innovative advances in modern farm management can resemble the notion of “digital twins”, which is the confluence of IoT, AI, and big data. A digital twin is “a digital replica of a living or non-physical entity” that is used “to create living digital simulation models that update and change as their physical counterparts change". In terms of farm management, digital twins mean that “farm operations no longer need physical proximity, for the remote monitoring, control, and coordination of farming operations"
RQ3	Among other positive impacts, SPACE collaborates to increase yields and the sustainability of agricultural systems
Case study 11	[59]
RQ1	An approach was proposed for creating a digital wheat twin based on multi-agent knowledge bases and technologies to model wheat cultivation. The need to develop physical cybernetic systems for the management of agricultural enterprises was discussed, providing the problem statement for creating a control object model, i.e., digital twin plant that will do the research and determine the entire plant growth and development cycle, as well as the production plan for the enterprise. It provides an overview of existing approaches to the development of digital twins and proposes a new approach with ontological models and multi-agent systems. It describes a multi-agent system for planning and modelling plant development, which is the core part of the plant’s digital twin. I discusses the ontology development and knowledge base of plant developmental stages, which are the basis for the digital twin and the interaction protocol between the agronomist and the digital twin plant
RQ2	Cyberphysical systems are a new type of system that integrate computing, communication, and control components, including sensors, actuators, and network connectors. Modern precision agriculture technologies with daily controlled plant cultivation can significantly improve product quality and agricultural production efficiency. This approach hypothesises that the reasoning of agronomists and other experts in farm management can be modelled as a self-organising process from the above entities, which can be implemented using multi-agent, plant-growing technology to simulate prospective scenarios for new crops, predicting returns and risks for the business. Furthermore, they can improve the quality and efficiency of agricultural management decisions
RQ3	The plant’s digital twin will be created for each field to mirror the plant’s current growth and development. It would mirror the daily development of the plant, representing the most anticipated version of the plant development plan, updated daily with data from the weather server, sensors in the fields, and observations from agronomists. Thus, before the agronomist makes suggestions about the actions that need to be taken in each field in a given situation, they can “simulate” the impact on the crop and analyse the possible “response”. This process needs to be supported in the proposed system so that the agronomist can use it, and compare it with the real plant response. In this way, knowledge about plant cultivation can be adjusted year after year, modifying the plant’s decision-making model and creating a more accurate digital twin, expanding a possible state graph of the agent under various conditions. The main idea of the proposed approach is to consider crop cultivation as a complex adaptive system with collective decisions distributed among crop varieties, soil, fertilisers, precise machines, etc.

## Data Availability

Not applicable.

## Abbreviations

The following abbreviations are used in this manuscript:

ACM	Association for Computing Machinery
DT	Digital Twin
DQ	Data extraction Question
EC	Exclusion Criteria
IC	Inclusion Criteria
IEEE	Institute of Electrical and Electronics Engineers Internet of Things
IoT	Internet of Things
PICOC	Population, Intervention, Comparison, Outcomes, Context
PRISMA	Preferred Report Items for Systematic reviews and Meta-analyses
QQ	Quality Question
SLR	Systematic Literature of Review
RQ	Research Question
SL	Springer Link
WoF	Web of Science
